# Identification of a Novel Pyrrole Alkaloid from the Edible Mushroom *Basidiomycetes-X* (Echigoshirayukidake)

**DOI:** 10.3390/molecules25214879

**Published:** 2020-10-22

**Authors:** Toshio Sakamoto, Ayaka Nishida, Naoki Wada, Yutaka Nakamura, Shinji Sato, Tetsuya Konishi, Seiichi Matsugo

**Affiliations:** 1 School of Biological Science and Technology, College of Science and Engineering, Kanazawa University, Kakuma, Kanazawa 920-1192, Japan; naoki-wada@se.kanazawa-u.ac.jp; 2 Division of Natural System, Graduate School of Natural Science and Technology, Kanazawa University, Kakuma, Kanazawa 920-1192, Japan; anrn019@gmail.com (A.N.); matsugoh@staff.kanazawa-u.ac.jp (S.M.); 3 Faculty of Applied Life Sciences, Niigata University of Pharmacy and Applied Life Sciences, Higashijima, Akiha-ku, Niigata 956-8603, Japan; nakamura@nupals.ac.jp (Y.N.); sato@nupals.ac.jp (S.S.); t.konishi@bg.wakwak.com (T.K.); 4 Office HALD Food Function Research, Inc., Yuzawa, Niigata 949-6102, Japan

**Keywords:** dietary supplement, edible fungus, pyrrole alkaloid

## Abstract

Three pyrrole alkaloid derivatives were isolated from the edible mushroom *Basidiomycetes-X* (Echigoshirayukidake) by water extraction followed by ethyl acetate fractionation. The chemical structures determined by MS and NMR were 4-[2-formyl-5-(hydroxymethyl)-1*H*-pyrrol-1-yl] butanoic acid (compound I), 4-[2-formyl-5-(hydroxymethyl)-1*H*-pyrrol-1-yl] butanamide (compound II), and 5-(hydroxymethyl)-1*H*-pyrrole-2-carboxaldehyde (compound III). Compound I was found to be the major component, followed by compound II, and compound III was the minor component. The dry powder of *Basidiomycetes-*X contained approximately 825 μg g^−1^ compound I and 484 μg g^−1^ compound II. Compound II was found to be a novel pyrrole aldehyde homologue not previously reported and thus is a specific component of this mushroom.

## 1. Introduction

Edible mushrooms are a well-known healthy food that have unique nutritional properties in that they are low calorie but rich in nutrients such as vitamins and minerals. Additionally, food factors such as indigestible polysaccharides as dietary fiber and other pharmacologically active ingredients are generally found in mushrooms [1]. Therefore, the search of new mushroom resources might be an important field of health sciences. *Basidiomycetes*-*X* (Japanese vernacular name: Echigoshirayukidake) is a novel edible mushroom found in the mountainous region of Niigata, Japan and was registered to the database of the NPO organization for International Patent Organism Depositing (IPOD) in the Industrial Technology Institute of Japan (PCT/JP2004/006418) in 1999 as a new species which belongs to Basidiomycetes family but uniquely does not form basidium. The artificially cultured mycelium mass (sclerotia) is now commercially available as the food and the medicinal resource, and, thus, the functional studies on this novel mushroom is currently progressed [2]. It has been reported that the aqueous extract of *Basidiomycetes*-*X* showed a potential hydroxylradical scavenging activity and protective effects on lipopolysaccharide-induced hepatic oxidative damage in mice [3]. Further studies revealed that *Basidiomycetes-X* has potential anti-obesity and hepatoprotective functions in rodents [4,5], and it inhibits atopic dermatitis in a mouse model [6]. Quite recently, *Basidiomycetes*-*X* was also reported to prevent and ameliorate non-alcoholic steatohepatitis (NASH) in fatty liver model rats [7]. Therefore, the active ingredients functioning in these activities wait to be clarified. In the present study, the candidate active components of *Basidiomycetes*-*X* were isolated by the separation of ethyl acetate soluble components from the aqueous extract of *Basidiomycetes*-*X* dried powder, since the ethyl acetate fraction showed high DPPH radical scavenging activity, Fe^3+^-reducing ability, and Cu^2+^-reducing ability (PCT/JP2018/043401). The chemical structures of the purified compounds were determined by MS and NMR.

## 2. Results

### Identification of Pyrrole Alkaloid Derivatives

The aqueous extracts of *Basidiomycetes*-*X* were analyzed by HPLC as described in the Materials and Methods section. Compound I at a retention time (Rt) of 36.8 min was detected as the major component in the HPLC chromatogram recorded by the absorbance at 260 nm, which accounted approximately 26% of the total observable peak area on the chromatogram. Compound I showed a characteristic UV absorption spectrum with an absorption maximum at 297 nm and a shoulder at 260 nm recorded by a photodiode array (PDA) detector. Compound II at a Rt of 30.2 min was detected as the second major component, which accounted approximately 21% of the total peak area on the chromatogram. Compound III was barely detectable as a minor peak at 25 min. Both compound II and compound III showed similar or almost identical absorption spectra to that of compound I, which suggests that these compounds have the same chromophore structure. Compounds I, II, and III were found to be ethyl acetate extractable components in the aqueous extracts of *Basidiomycetes*-*X.* Thus, these compounds were further purified, and their chemical structures were determined as described in the Materials and Methods section.

**Figure 1:**
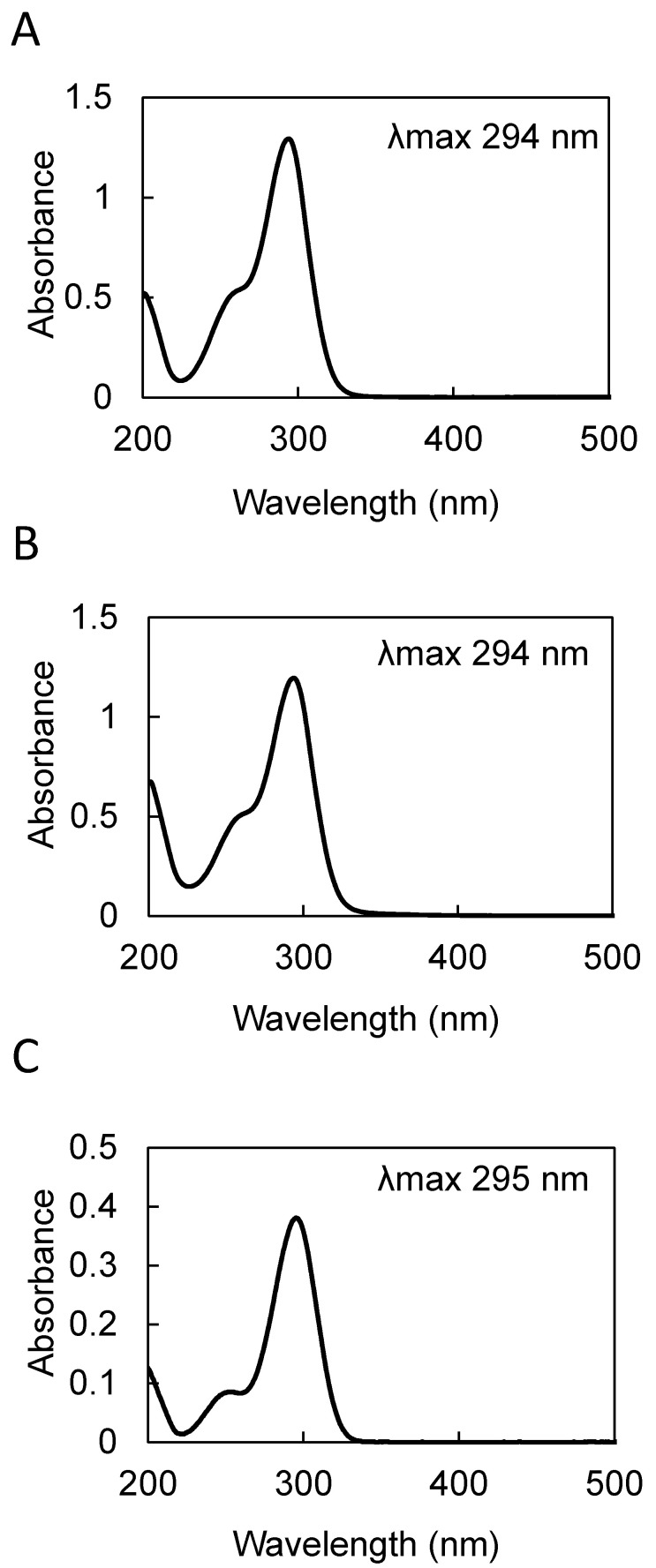
UV-VIS absorption spectra of the pyrrole alkaloid derivatives purified from *Basidiomycetes-X* in methanol. (**A**) 4-[2-formyl-5-(hydroxymethyl)-1*H*-pyrrol-1-yl] butanoic acid. (**B**) 4-[2-formyl-5-(hydroxymethyl)-1*H*-pyrrol-1-yl] butanamide. (**C**) 5-(hydroxymethyl)-1*H*-pyrrole-2-carboxaldehyde.

**Figure 2:**
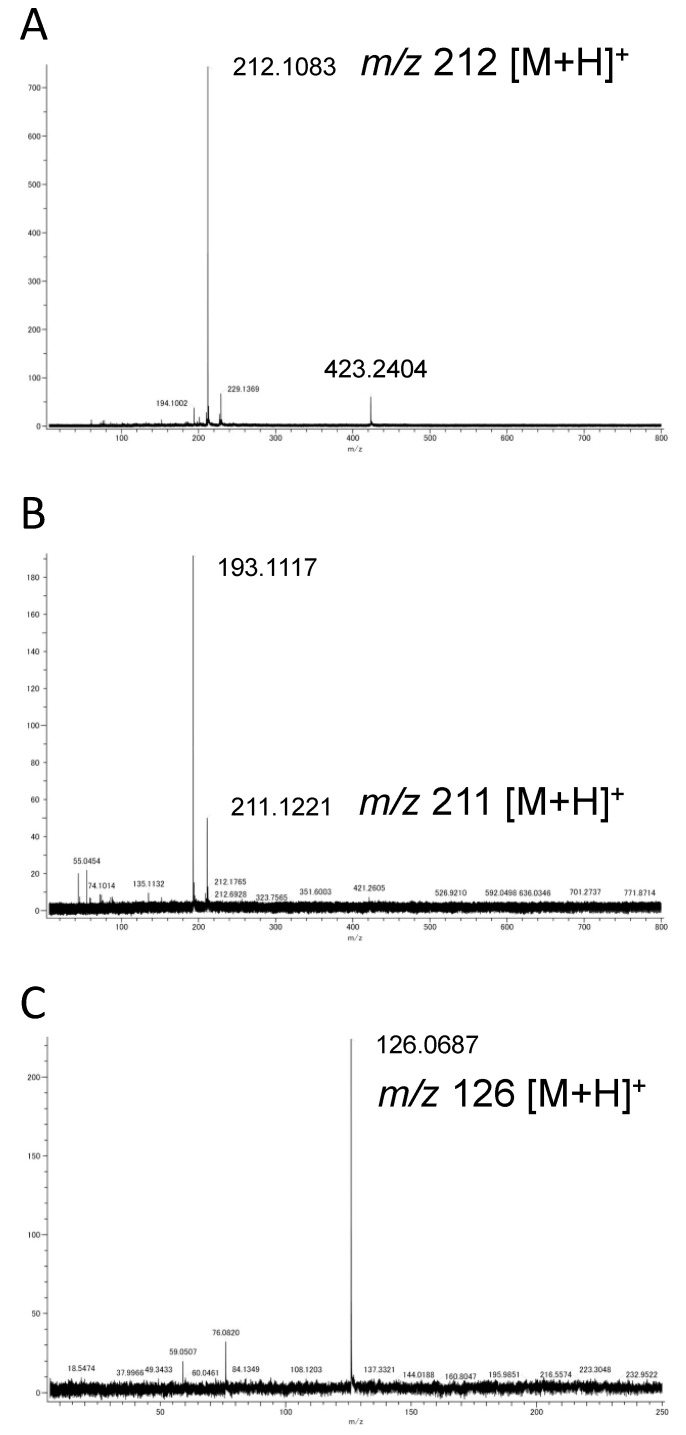
DART-MS spectra of the pyrrole alkaloid derivatives purified from *Basidiomycetes-X*. (**A**) 4-[2-formyl-5-(hydroxymethyl)-1*H*-pyrrol-1-yl] butanoic acid. (**B**) 4-[2-formyl-5-(hydroxymethyl)-1*H*-pyrrol-1-yl] butanamide. (**C**) 5-(hydroxymethyl)-1*H*-pyrrole-2-carboxaldehyde.

**Figure 3:**
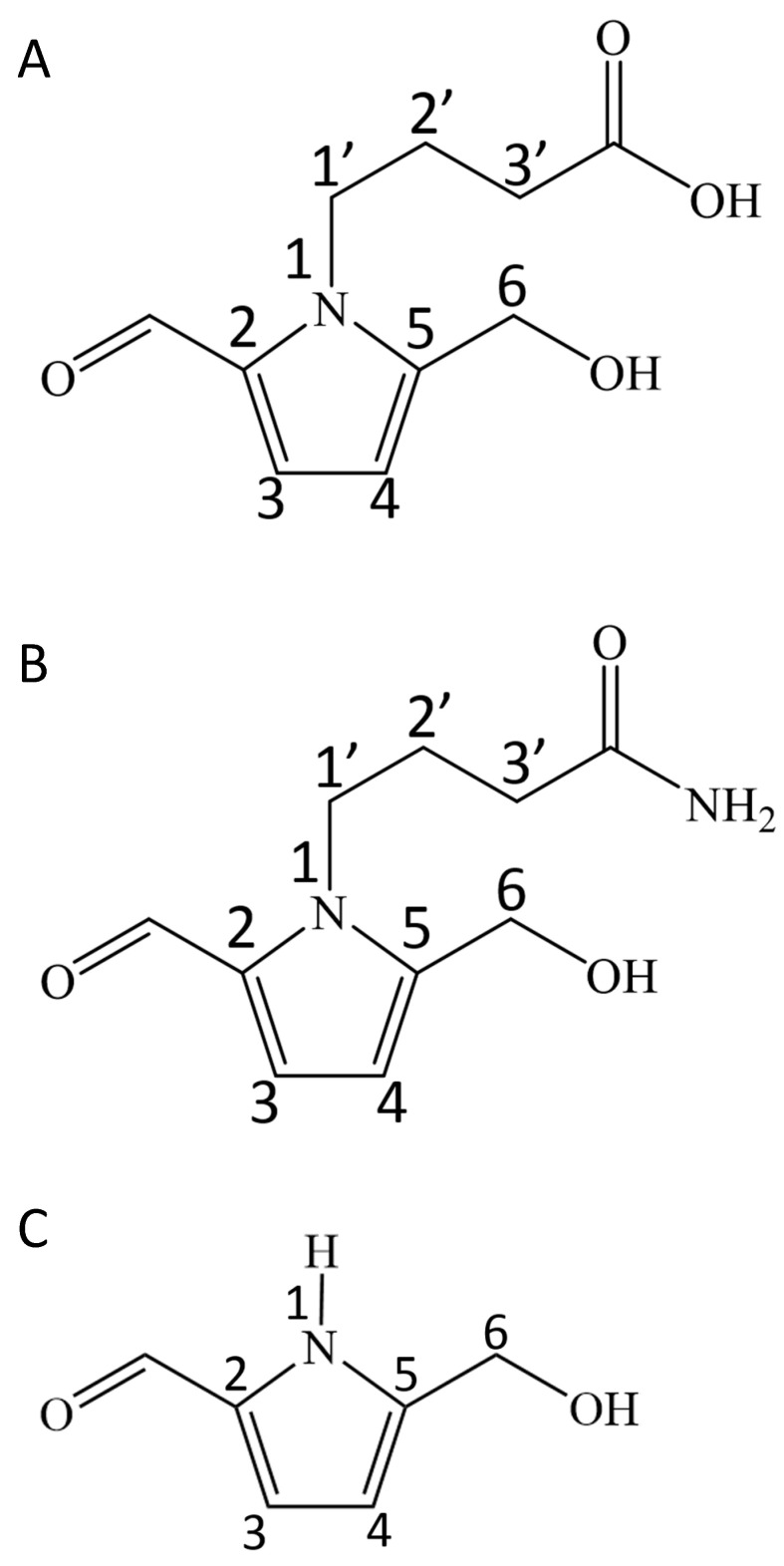
Structures of the pyrrole alkaloid derivatives from *Basidiomycetes-X*. (**A**) 4-[2-formyl-5-(hydroxymethyl)-1*H*-pyrrol-1-yl] butanoic acid. (**B**) 4-[2-formyl-5-(hydroxymethyl)-1*H*-pyrrol-1-yl] butanamide. (**C**) 5-(hydroxymethyl)-1*H*-pyrrole-2-carboxaldehyde.

Purified compound I showed a characteristic UV absorption spectrum with an absorption maximum at 294 nm and a shoulder at 260 nm (Figure 1A). The molecular mass determined by direct analysis in real time mass spectrometry (DART-MS) analysis was 211 (Figure 2A). The formula of compound I was estimated by fast atom bombardment high resolution mass spectrometry (FAB-HR-MS) analysis and was determined to be C_10_H_14_NO_4_ (Table 1). Table 2 shows the summary of the ^13^C and ^1^H NMR analysis of compound I (Supplementary Materials), and the data was essentially identical to the previously reported data [8]. Taken together, these results identified compound I as 4-[2-formyl-5-(hydroxymethyl)-1*H*-pyrrol-1-yl] butanoic acid (Figure 3A).

Purified compound II showed a UV absorption spectrum with an absorption maximum at 294 nm and a shoulder at 260 nm (Figure 1B), similar to that of the compound I (Figure 1A). The molecular mass of compound II determined by DART-MS analysis was 210 (Figure 2B), and its formula predicted by FAB-HR-MS analysis was C_10_H_15_N_2_O_3_ (Table 1). Table 3 shows the summary of the ^13^C and ^1^H NMR analysis of compound II. The ^13^C signal at 175.6 ppm and the ^1^H signals at 6.23 ppm and 5.67 ppm indicate the presence of the amide group, which is a characteristic feature of compound II. In summary, these results identified compound II as 4-[2-formyl-5-(hydroxymethyl)-1*H*-pyrrol-1-yl] butanamide (Figure 3B), which is an amide derivative of compound I.

Purified compound III showed a UV absorption spectrum with an absorption maximum at 295 nm and a shoulder at 260 nm (Figure 1C). The molecular mass of compound III determined by DART-MS analysis was 125 (Figure 2C), and its predicted formula determined by FAB-HR-MS analysis was C_6_H_8_NO_2_ (Table 1). Table 4 shows the summary of the ^1^H NMR analysis of the compound III. The data were basically identical to the data previously reported [9]. Taken together, these results identified compound III as 5-(hydroxymethyl)-1*H*-pyrrole-2-carboxaldehyde (Figure 3C).

Table 5 shows the contents of the pyrrole alkaloid derivatives detected in the dried powder of *Basidiomycetes*-*X*. The calculated yields were 2.3% for compound I, 2.9% for compound II, and 10% for compound III, respectively, when extracted from 50 g of the dry powder as the starting material.

## 3. Discussion

Currently pyrrole aldehyde derivatives are identified in many plant and fungal resources as the ingredients having medicinal functions [10]. In the present study, we determined the pyrrole alkaloid derivatives in *Basidiomycetes-X* (Echigoshirayukidake) mushroom, which is a newly found Basidiomycota family mushroom in Niigata, Japan [2]. Among the three pyrrole aldehyde homologues identified, compound I was the most abundant, followed by compound II. Compound III was only a trace (Table 5). Moreover, compound II (butamide derivative) (Figure 3B) was the novel analogue of pyrrole aldehydes thus far reported and was assigned as the specific analogue of *Basidiomycetes*-*X*. In contrast, compounds I and III were reported as active ingredients in the fruits of *Lycium chinense* [8] and *Morus alba* [11] that have hepatoprotective and macrophage activation functions, respectively. Compound I is also found in mushrooms such as *Leccinum extremiorientale* [12] and *Inonotus obliquus* [13]. Recently, the same types of pyrrole alkaloid derivatives have been isolated from the fruiting bodies of another edible mushroom *Phlebopus portentosus* [14], and it has been reported that they significantly attenuated H_2_O_2_-induced cell damage in human neuroblastoma SH-SY5Y cells [14]. We have been pursuing antioxidants in the aqueous extracts of *Basidiomycetes*-*X*, however, the purified compound II displayed very weak DPPH radical scavenging activity (data not shown). Antioxidant activity guided approach to seek the active components from *Basidiomycetes-X* is ongoing and will be reported in the future.

The present finding that the formyl pyrrole alkaloids are present in *Basidiomycetes-X,* therefore, supports the idea that these pyrrole alkaloids are playing crucial roles in the observed medicinal and pharmacological functions of this mushroom so far, we reported elsewhere [3,4,5,6,7]. Especially, the finding of amide analogue (compound II) as the novel ingredient of *Bashidiomycetes*-X provided a new insight to the functions of this mushroom because the amide structure, which is rather stable, will differentiate the metabolic behaviors from the acid homologue (compound I), and, thus, a discussion will be expanded if they have similar functions or some new functions are involved in the amide analogue. Further mechanistic studies on this unique ingredient will establish further background for the potential importance of this novel mushroom as a resource for dietary supplements or functional food development. Experiments of oral administration to animals are planned to evaluate the biological activities using the chemically synthesized compounds I, II, and III, and the precise results will be reported elsewhere.

Since the formyl pyrrole structure is formed from d-glucose and γ-aminobutyric acid via the Maillard reaction [10,15], there is some discussion on whether these attractive ingredients are produced chemically or by a biosynthetic route. The present study further supported that the formyl pyrrole ingredients are generally found in a variety of natural resources and, thus, further studies on the biosynthetic mechanisms are needed for clarification.

## 4. Materials and Methods

### 4.1. Purification of Pyrrole Alkaloids

The dry powder of the edible mushroom *Basidiomycetes-X* (Echigoshirayukidake) was provided by Mycology Techno Co., Ltd. (Niigata, Japan). *Basidiomycetes-*X powder (50 g) was suspended in 500 mL of distilled water and stirred for 24 h at room temperature. After filtration through a filter paper, the filtrate (430 mL) was collected, and 160 mL of ethanol was added. After centrifugation at 21,500 × *g* for 15 min at room temperature to remove the precipitates, the supernatant (540 mL) was recovered and divided into 135 mL aliquots. Ethyl acetate (90 mL) was added to the 135-mL supernatant and vigorously mixed using a 500-mL separatory funnel. Then, the upper organic phase was recovered. The ethyl acetate extraction was repeated twice for each 135-mL fraction, and the organic solution (920 mL in total) was collected. After removing the solvent using a rotary evaporator, the residue was dissolved in 100% methanol (8 mL). The methanol-soluble sample was applied onto a silica gel plate (PLC Silica gel 60 F_254_, 2 mm, 20 by 20 cm, Merck Millipore, Darmstadt, Germany) and developed by chloroform-methanol (6:4, *v*/*v*) as a solvent. After drying the plate, UV absorbing compounds were detected by UV illumination at 254 nm. The band at a retardation factor (Rf) value of 0.56 (band I) and the band at a Rf value of 0.88 (band II) were collected separately. After grinding the silica gel into powder, the samples were dissolved in methanol. The methanol extracts were filtered through filter paper and successively through a 0.45-μm polytetrafluoroethylene (PTFE) syringe filter (RJF1345NH, RephiLe Bioscience, Ltd., Boston, MA, USA). After evaporation using a rotary evaporator, the residues derived from band I (41.9 mg) and band II (82.6 mg) were separately dissolved by 40% (*v*/*v*) and by 30% (*v*/*v*) methanol, respectively, and further fractionated using the preparative HPLC system described below.

The 40% methanol-soluble sample from band I was filtered through a 0.45-μm PTFE syringe filter (RJF1345NH, RephiLe), subjected to HPLC analysis using a reverse phase column (Inertsustain, 5 μm, 14 by 150 mm, GL Sciences Inc., Tokyo, Japan) and separated using 40% methanol with 0.1% acetic acid (*v*/*v*) as the mobile phase at a flow rate of 5.5 mL min^−1^. The eluted peaks were monitored by the absorbance at 260 nm, and the fraction at a Rt of 6.3 min was collected, evaporated and dissolved in 100% methanol. The yield of the purified fraction was 7.5 mg, and it was used for the structural analysis as compound I.

The 30% methanol-soluble sample from band II was filtered through a 0.45-μm PTFE syringe filter (RJF1345NH, RephiLe), subjected to a HPLC system equipped with a reverse phase column (Inertsustain, 5 μm, 14 by 150 mm, GL Sciences), and then separated using a mobile phase of 30% methanol with 0.1% acetic acid (*v*/*v*) at a flow rate of 5.5 mL min^-1^. The A_260_ was used for monitoring the peaks. The fraction at a Rt of 7.0 min was collected, evaporated and dissolved in 100% methanol. The purified fraction with a 6.3 mg yield was used for the structural analysis as compound II.

Band III at a Rt of 5.9 min was also recovered with 30% methanol, evaporated and redissolved in 20% methanol (*v*/*v*). The sample was further purified using the same HPLC system as above except using 20% methanol with 0.1% acetic acid (*v*/*v*) as the mobile phase. The peak at a Rt of 8.8 min was collected, evaporated, and dissolved in 100% methanol. The yield of purified band III was 0.5 mg, and it was used for the structural analysis as compound III.

### 4.2. Analytical HPLC System

The aqueous extracts of *Basidiomycetes*-*X* were analyzed by HPLC and the purity was assessed during the preparation of the pyrrole alkaloids. After filtration through a 0.45-μm PTFE syringe filter (RJF1345NH, RephiLe), the sample was injected into a HPLC system (PU-2087 Plus Intelligent Prep. Pump, JASCO Co., Hachioji, Tokyo, Japany) equipped with a reverse phase column (Cosmosil 5C18-MS, 5 μm, 4.6 by 150 mm, Nacalai Tesque, Inc., Kyoto, Japan). The mobile phase was 0.1% acetic acid for the initial 5 min and changed linearly to methanol with 0.1% acetic acid for the next 95 min for gradient elution. The flow rate was constant at 0.6 mL min^-1^. The eluted peaks were monitored by a PDA detector (MD-2018 Plus Photodiode Array Detector, JASCO Co., Hachioji, Tokyo, Japan) to obtain the UV-VIS spectra.

### 4.3. MS Analysis

DART MS analysis was performed at the Research Institute for Instrumental Analysis in Kanazawa University using a mass spectrometer with a DART ionization module (JMS-T100TD; JEOL Ltd., Akishima, Tokyo, Japan). To predict the elemental composition, FAB HR-MS analysis was performed using the JEOL JMS-700 mass spectrometer using 3-nitrobenzyl alcohol (NBA) as a matrix.

### 4.4. Spectroscopic Analysis

The UV-VIS spectra were recorded using a Hitachi U-3900 spectrophotometer (Hitachi High-Tech Science Co., Tokyo, Japan). NMR spectra were measured by a JEOL ECA-600 or JEOL ECS400 spectrometer (JEOL Ltd., Akishima, Tokyo, JAPAN) at the Research Institute for Instrumental Analysis in Kanazawa University. 3-(Trimethylsilyl)-1-propanesulfonic acid-d6 sodium salt (TMP) was used as an internal NMR standard.

### 4.5. Measurement of the Pyrrole Alkaloid Contents by HPLC

*Basidiomycetes-*X powder (1 g) was suspended in 10 mL of 50% methanol and extracted by string for 24 h at room temperature. After centrifugation at 21,000× *g* for 2 min, the supernatant was filtrated through a 0.45-μm PTFE syringe filter (RJF1345NH, RephiLe). The sample (20 μL) was injected into the analytical HPLC system described above. Pyrrole alkaloid derivatives were identified by the characteristic UV absorption spectrum, and A_295_ was traced to draw the chromatogram.

The authentic standards of the pyrrole alkaloids I, II, and III were synthesized chemically at Niigata University of Pharmacy and Applied Life Sciences (Yutaka Nakamura, unpublished). Known amounts of the synthesized pyrrole alkaloids I (159 ng), II (412 ng), and III (137 ng) were injected into the HPLC system separately, and the amounts of the pyrrole alkaloids were calculated by comparing their peak area to that of the standards.

## 5. Patents

Matsugo, S.; Sakamoto, T.; Nishida, A.; Wada, N.; Konishi, T. Pyrrole Compound. PCT/JP2018/043401 11/26/2018

## Figures and Tables

**Table 1:** Summary of the FAB-HR MS analysis of the pyrrole alkaloid derivatives purified from *Basidiomycetes-X*.

Derivative	Observed *m*/*z*	Predicted Formula	Monoisotopic Mass	Error	
				ppm	mmu
Compound I ^a^	212.0913	C_10_H_14_NO_4_	212.0922	−4.6	−1.0
Compound II ^b^	211.1081	C_10_H_15_N_2_O_3_	211.1082	−0.8	−0.2
Compound III ^c^	126.0562	C_6_H_8_NO_2_	126.0555	+5.5	+0.7

^a^ 4-[2-formyl-5-(hydroxymethyl)-1*H*-pyrrol-1-yl] butanoic acid; ^b^ 4-[2-formyl-5-(hydroxymethyl)-1*H*-pyrrol-1-yl] butanamide.; ^c^ 5-(hydroxymethyl)-1*H*-pyrrole-2-carboxaldehyde.

**Table 2:** Summary of the NMR analysis of compound I.

Position	^13^C [ppm]	^1^H [ppm] (*J* in Hz)	HMBC (H to C)	^13^C [ppm] [8]	^1^H [ppm] (*J* in Hz) [8]
2	133.5	-	-	132.4	
3	126.4	6.99, d (4.12)	2, 4, 5, -CHO	124.7	6.98, d (4.1)
4	111.5	6.27, d (4.12)	2, 3, 5	110.8	6.26, d (4.1)
5	144.7	-	-	141.7	
6	56.4	4.64, s	4, 5	56.2	4.63, s
1′	45.8	4.40, t * (7.56)	2′, 3′, 2, 5	44.6	4.39, t (7.3)
2′	27.7	2.01, m	1′, 3′, -COOH	25.9	2.00, q (7.3)
3′	31.8	2.33, t (7.39)	1′, 2′, -COOH	30.2	2.31, t (7.3)
-CHO	180.9	9.42, s	2	179.6	9.41, s
-COOH	176.8	-	-	177.0	

NMR spectra were measured by a JEOL ECA600 spectrometer in methanol-d4 as a solvent; *: triplet-like coupling; Each proton and carbon signal was assigned from the correlation appeared in HMQC chart; The NMR spectroscopic data are essentially identical to those reported by Chin et al. [8]. The structure of compound I is shown in Figure 3A.

**Table 3:** Summary of the NMR analysis of compound II.

Position	^13^C [ppm]	^1^H [ppm] (*J* in Hz)	HMBC (H to C)
2	133.2	-	-
3	125.0	6.91, d (4.12)	2, 4, 5
4	111.0	6.21, d (4.12)	2, 3, 5
5	143.9	-	-
6	56.1	4.57, s	4, 5
1′	45.6	4.31, t * (7.33)	2′, 3′, 2, 5
2′	27.5	1.94, m **	1′, 3′, -CONH_2_
3′	32.4	2.22, t (7.20)	1′, 2′, -CONH_2_
-CHO	180.3	9.47, s	2
-CONH_2_	175.6	-	-
-NH_2_	-	6.23, brd s	-
	-	5.67, brd s	-

NMR spectra were measured by a JEOL ECS400 spectrometer in acetonitrile-d3 as a solvent. *: Triplet-like coupling. **: Multiplet peaks including solvent (CD_3_CN) peak. Each proton and carbon signal was assigned from the correlations appeared in HMQC chart. The structure determined for compound II is shown in Figure 3B.

**Table 4:** Summary of the NMR analysis of compound III.

Position	^1^H [ppm] (*J* in Hz)	^1^H [ppm] (*J* in Hz) [9]
2	-	
3	6.91, d (3.66)	6.96, dd (3.7, 3.0)
4	6.20, d (3.66)	6.20, dd (3.7, 2.2)
5	-	
6	4.56, s	4.81, s
-CHO	9.43, s	9.36, s
-OH	3.33, brd s	3.66, brd s
-NH	10.10, brd s	10.75, brd s

NMR spectra were recorded with a JEOL ECS400 spectrometer in acetonitrile-d3 as a solvent. Reference NMR spectrum was obtained in chloroform-d [9]. The structure of compound III is shown in Figure 3C.

**Table 5:** Contents of the pyrrole alkaloid derivatives in *Basidiomycetes-X.*

Derivative	Content
	μg [g DW]^−1^
Compound I ^a^	825 ± 39
Compound II ^b^	484 ± 23
Compound III ^c^	12 ± 1

Data are presented as the means ± SD (N = 3). The contents were determined by HPLC using the synthesized standards as described in the Materials and Methods section. ^a^ 4-[2-formyl-5-(hydroxymethyl)-1*H*-pyrrol-1-yl] butanoic acid. ^b^ 4-[2-formyl-5-(hydroxymethyl)-1*H*-pyrrol-1-yl] butanamide. ^c^ 5-(hydroxymethyl)-1*H*-pyrrole-2-carboxaldehyde.

## Supplementary Materials

The following are available online, NMR data of compounds I, II, and III.
