# Supplementary figures and images for: Identification of a Novel Pyrrole Alkaloid from the Edible Mushroom Basidiomycetes-X (Echigoshirayukidake)
Source: Molecules. 2020 Oct 22;25(21):4879. doi: 10.3390/molecules25214879 (PMC7672639; doi:10.3390/molecules25214879)

# Compound-I 1H-NMR

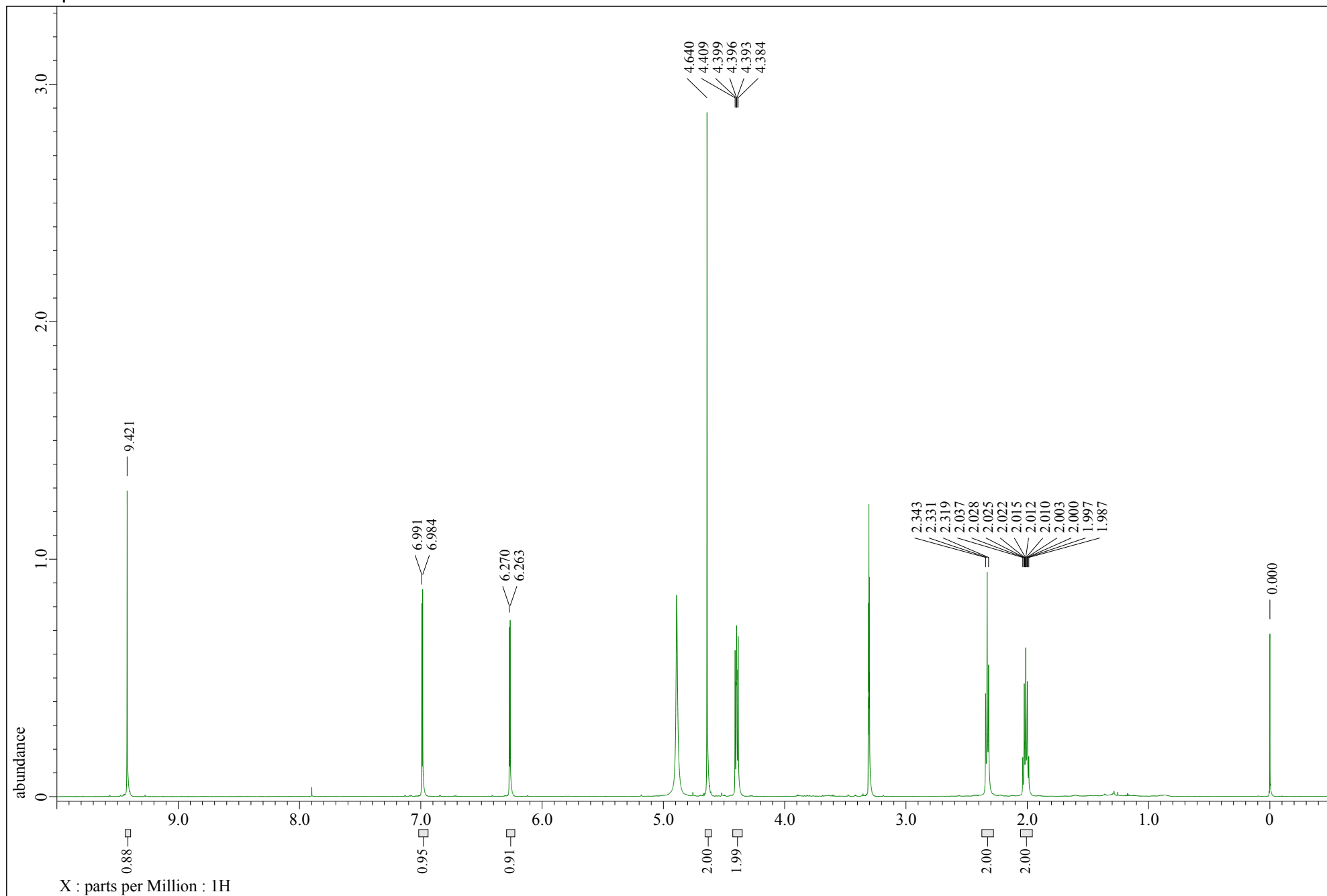

# <sup>1</sup>H-NMR 1-3ppm

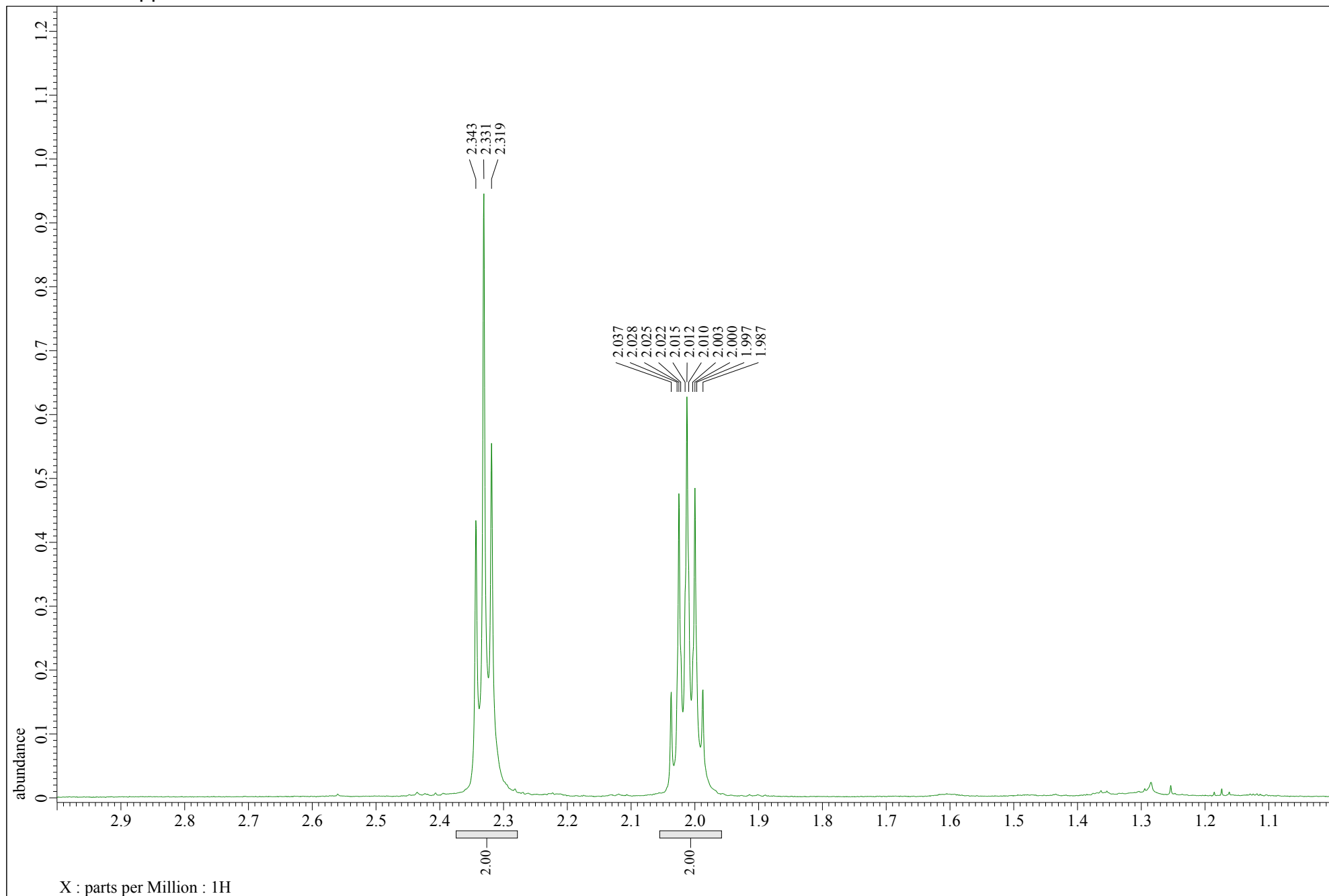

<sup>1</sup>H- NMR 4-5ppm

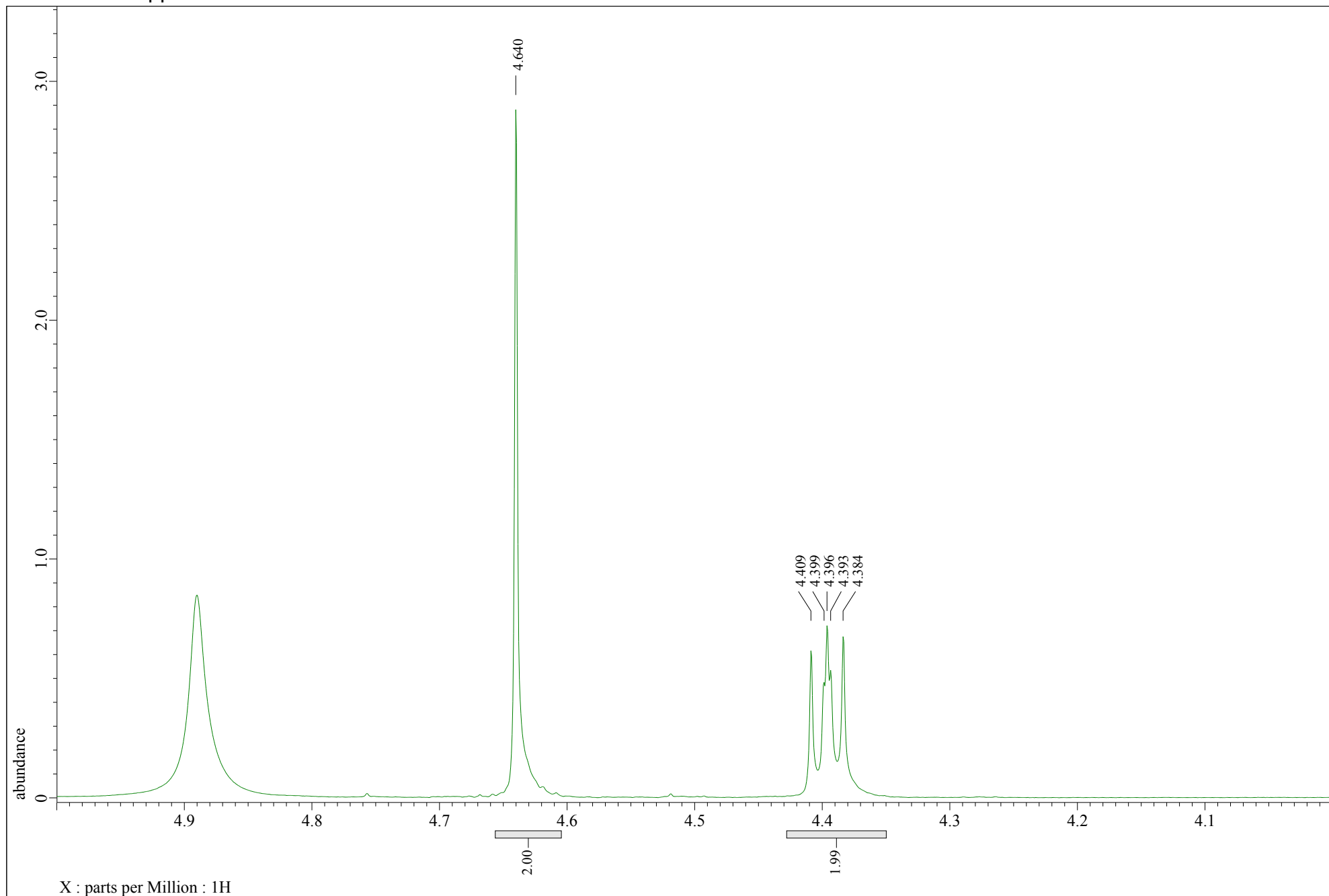

<sup>1</sup>H-NMR 5-7.5ppm

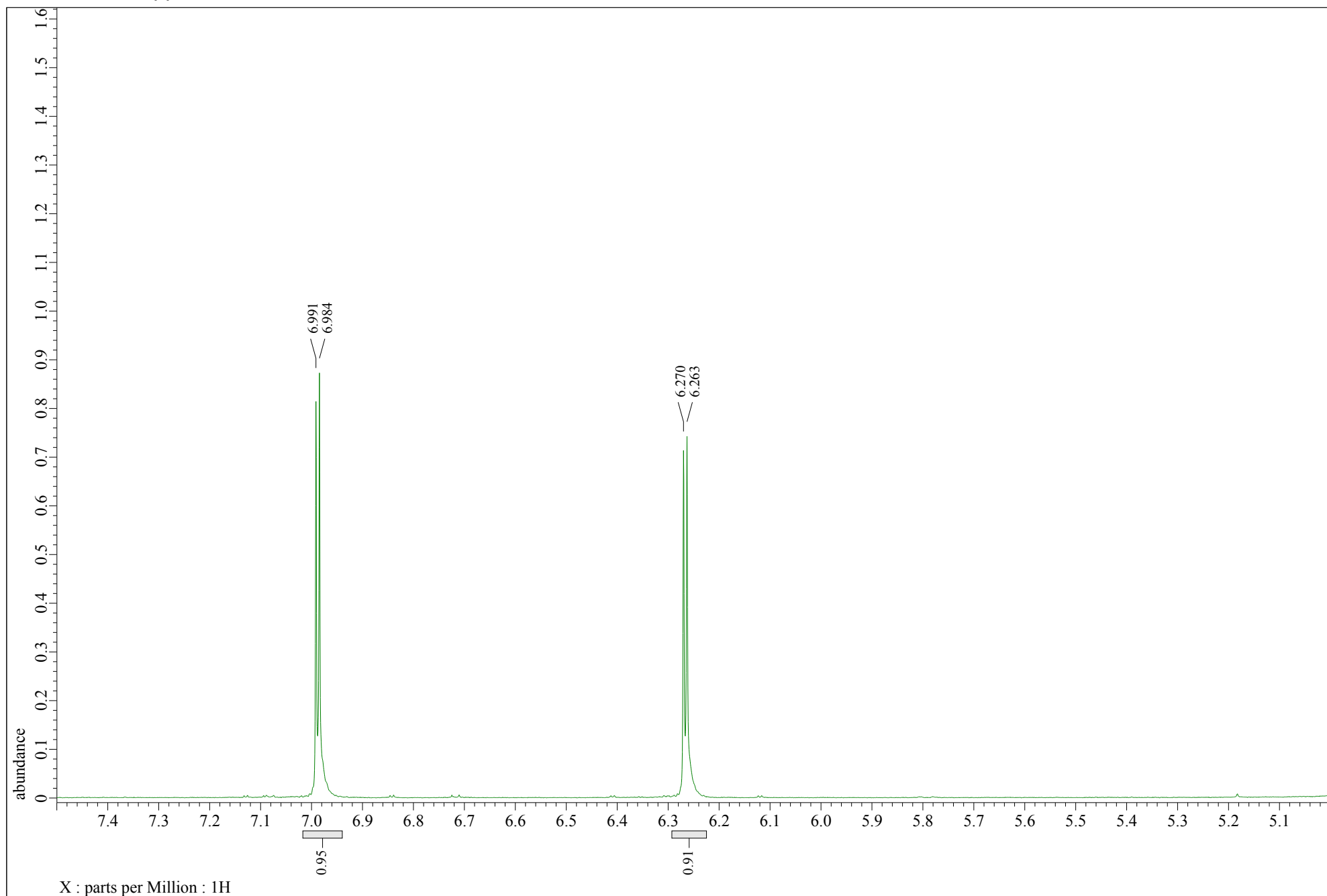

# <sup>13</sup>C-NMR

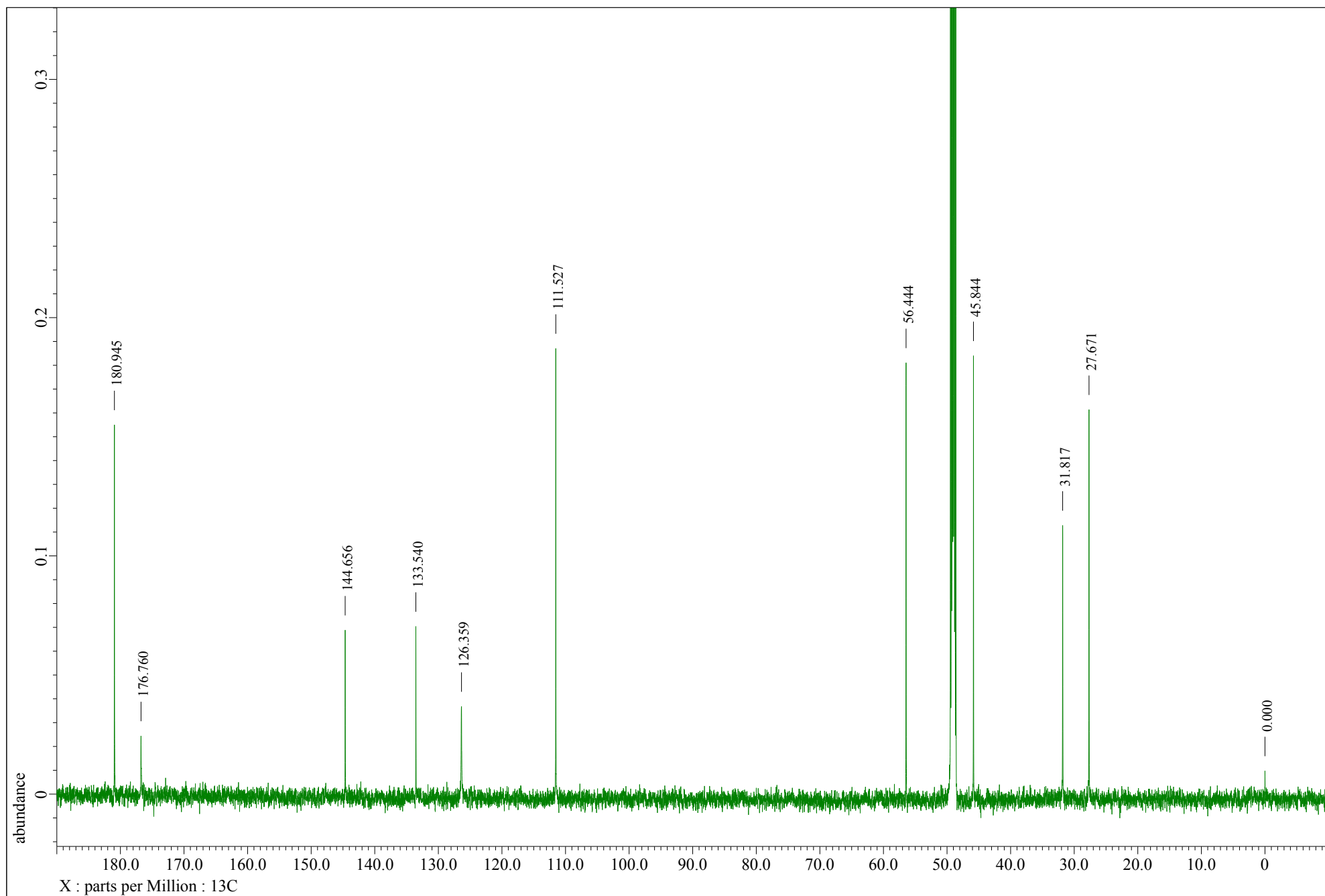

# HMQC

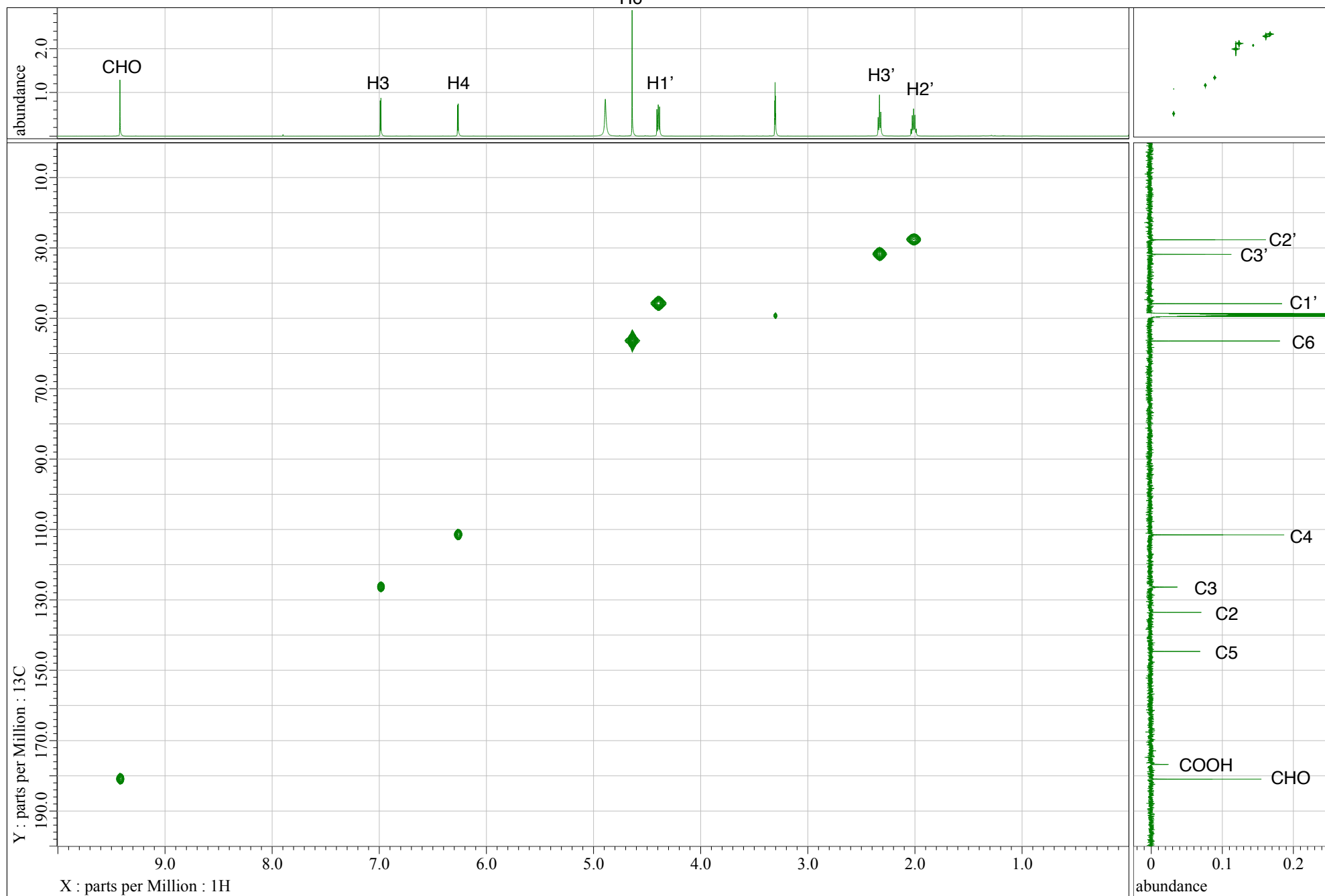

# HMBC(4 Hz)

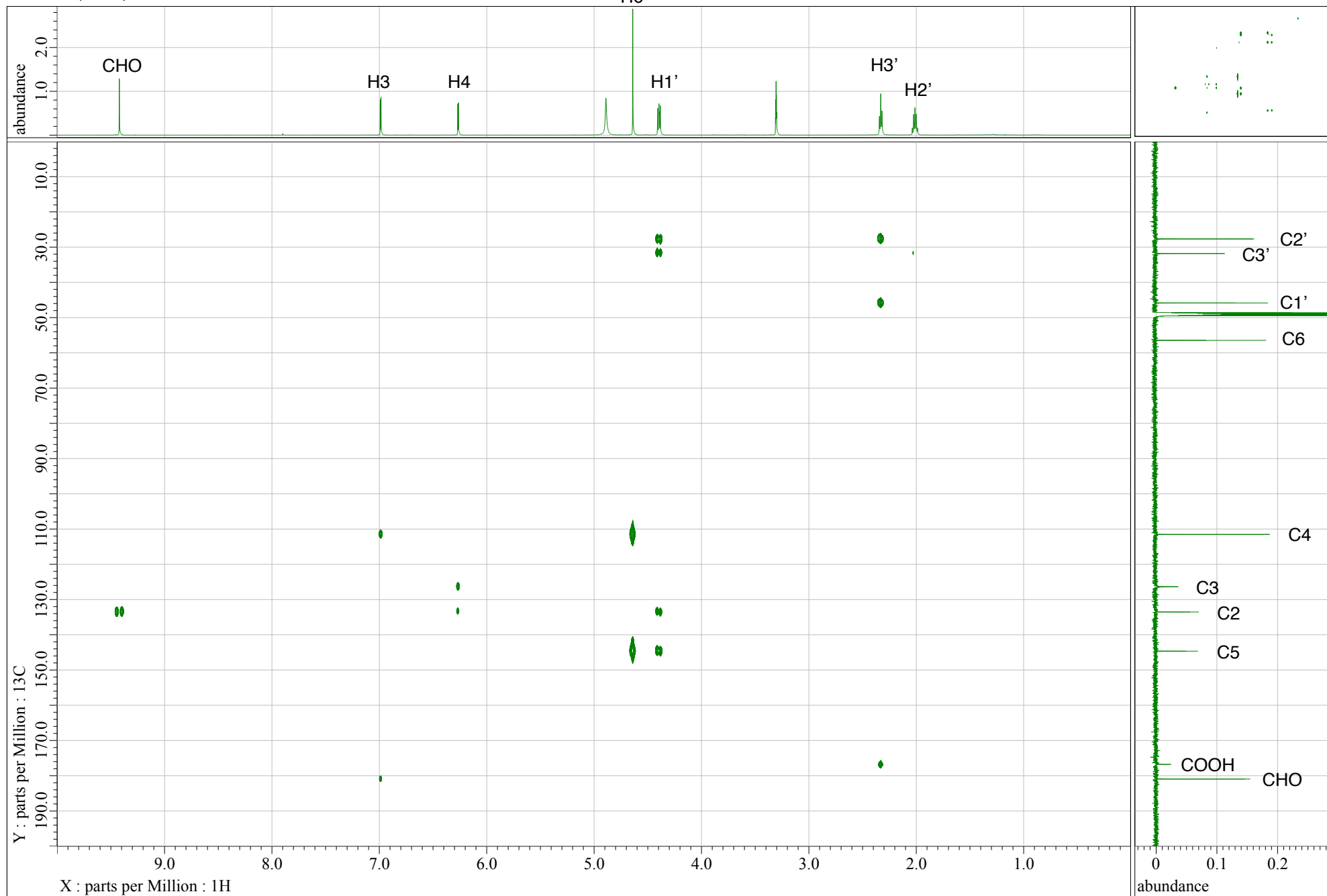

HMBC(8 Hz)

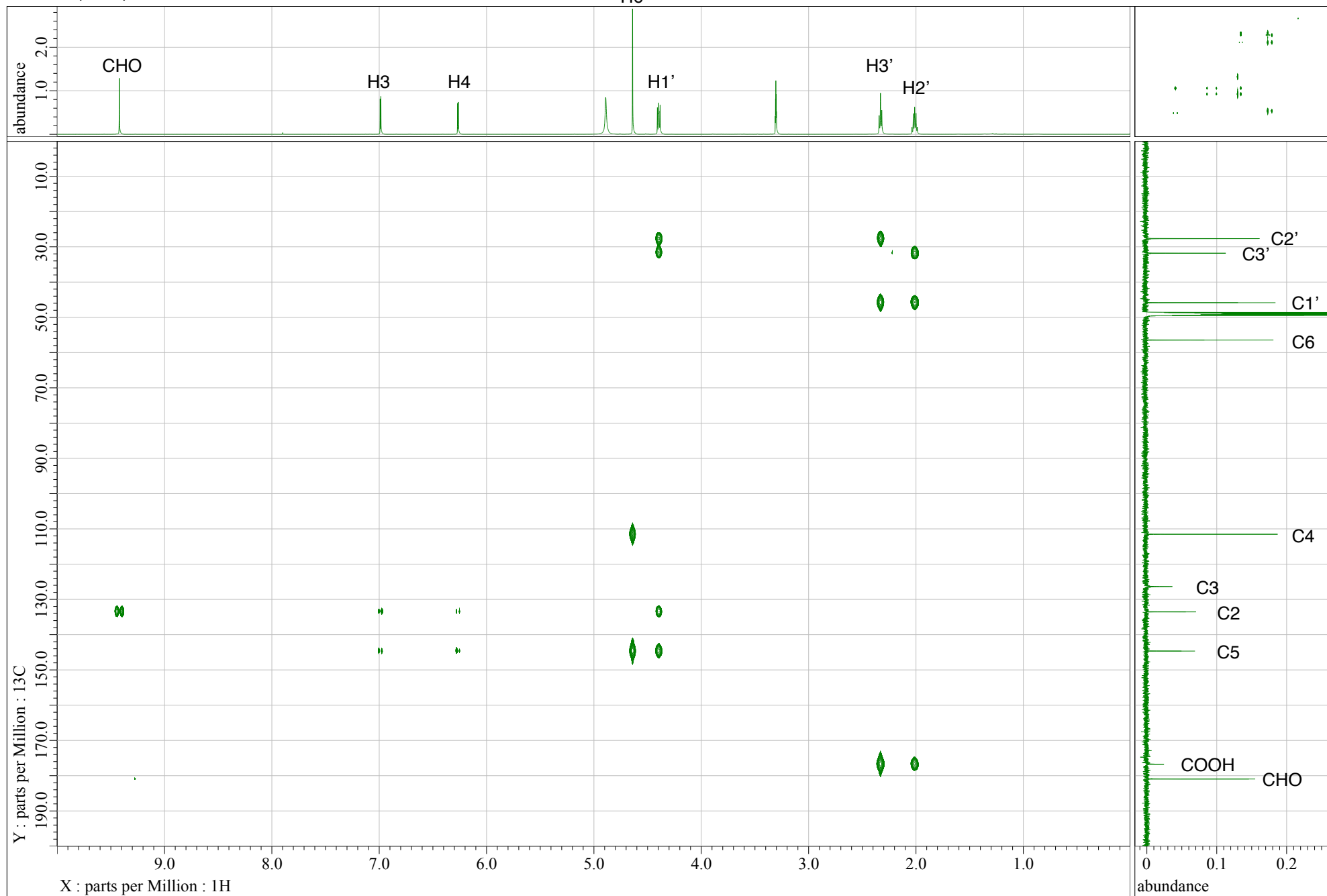

Supplement: Supplementary file 1 [file molecules-25-04879-s001.zip › NMR_data_r/Compound_I_NMR.pdf]

Compound-II 1H-NMR

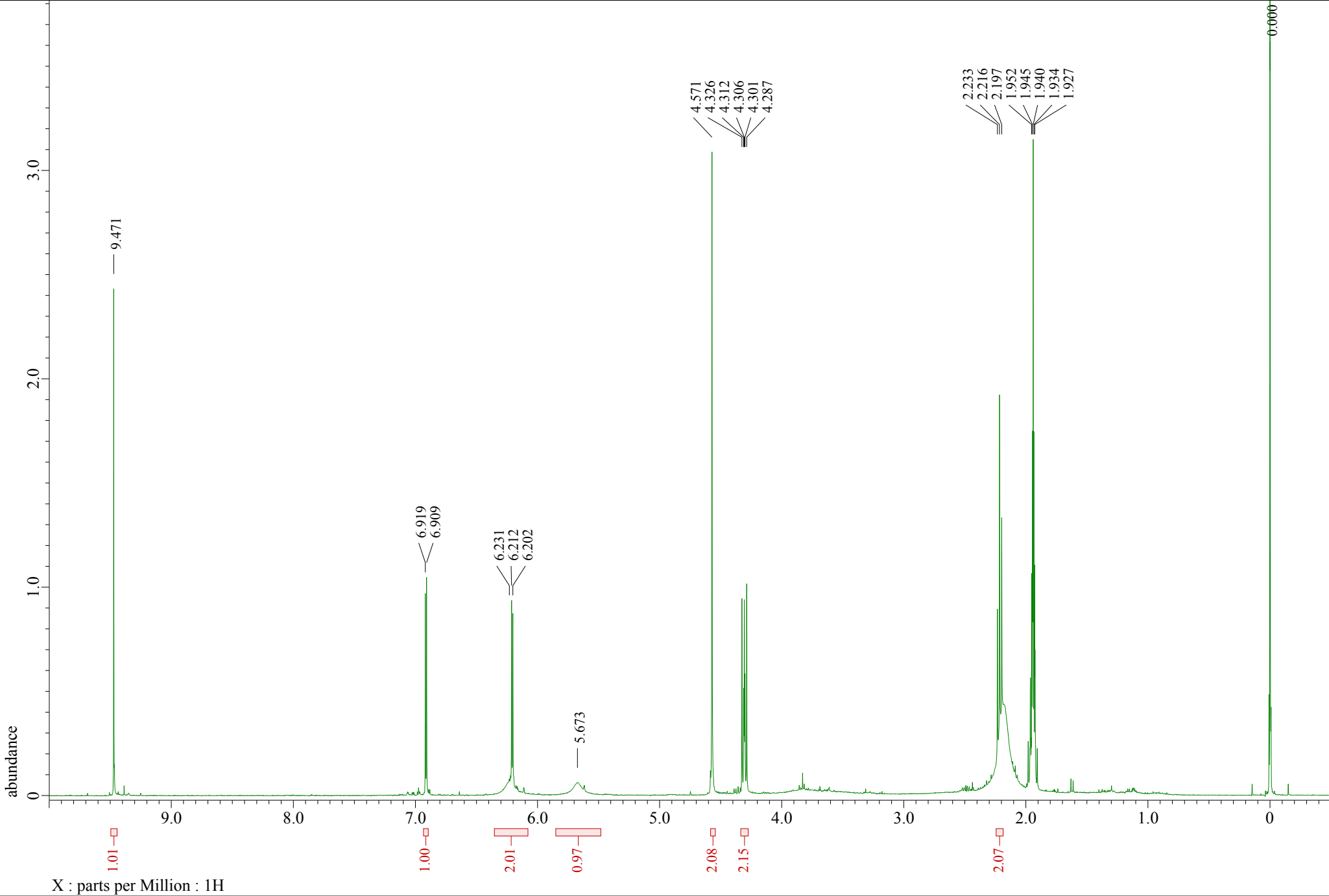

1H-NMR 1-3ppm

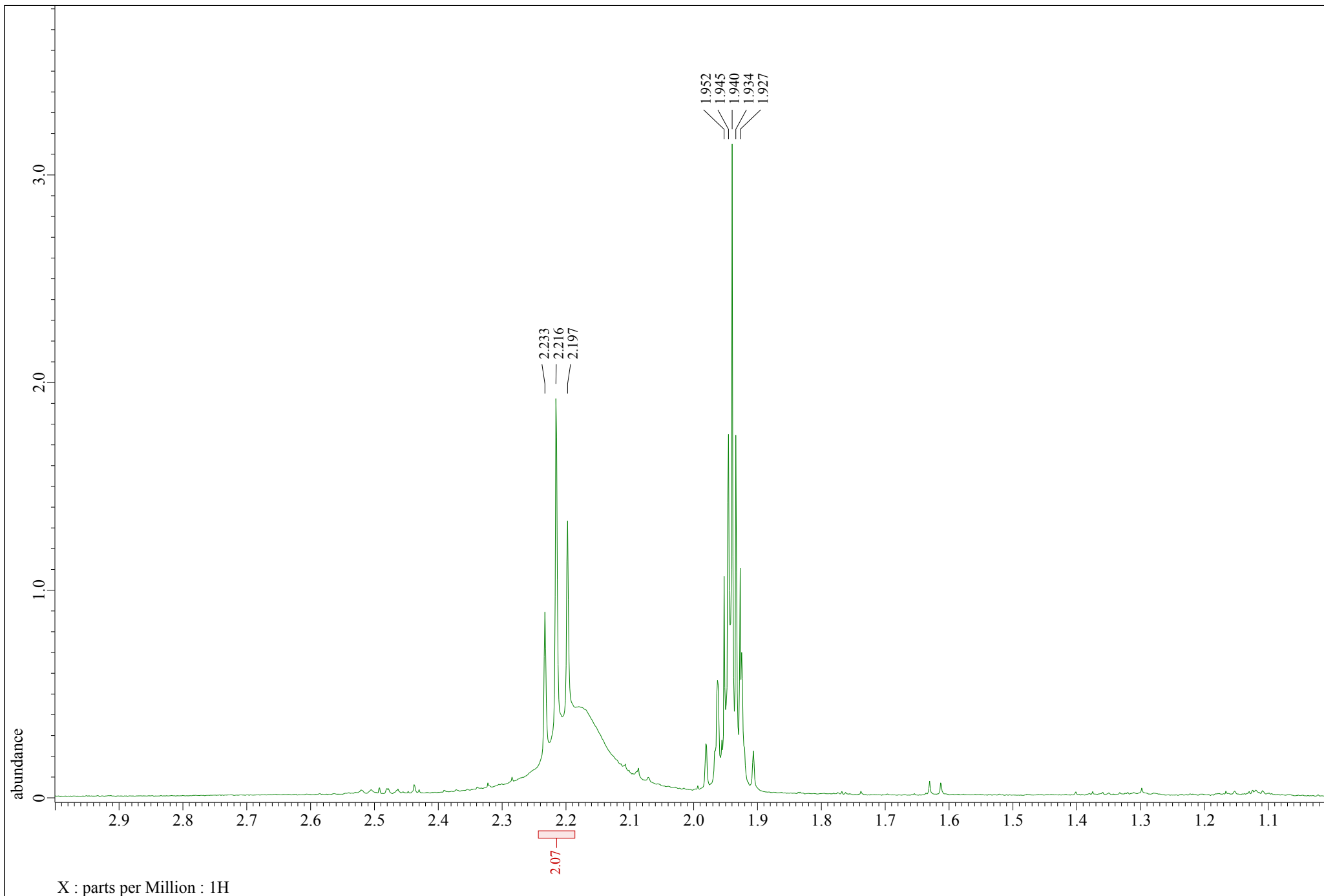

<sup>1</sup>H-NMR 4–5ppm

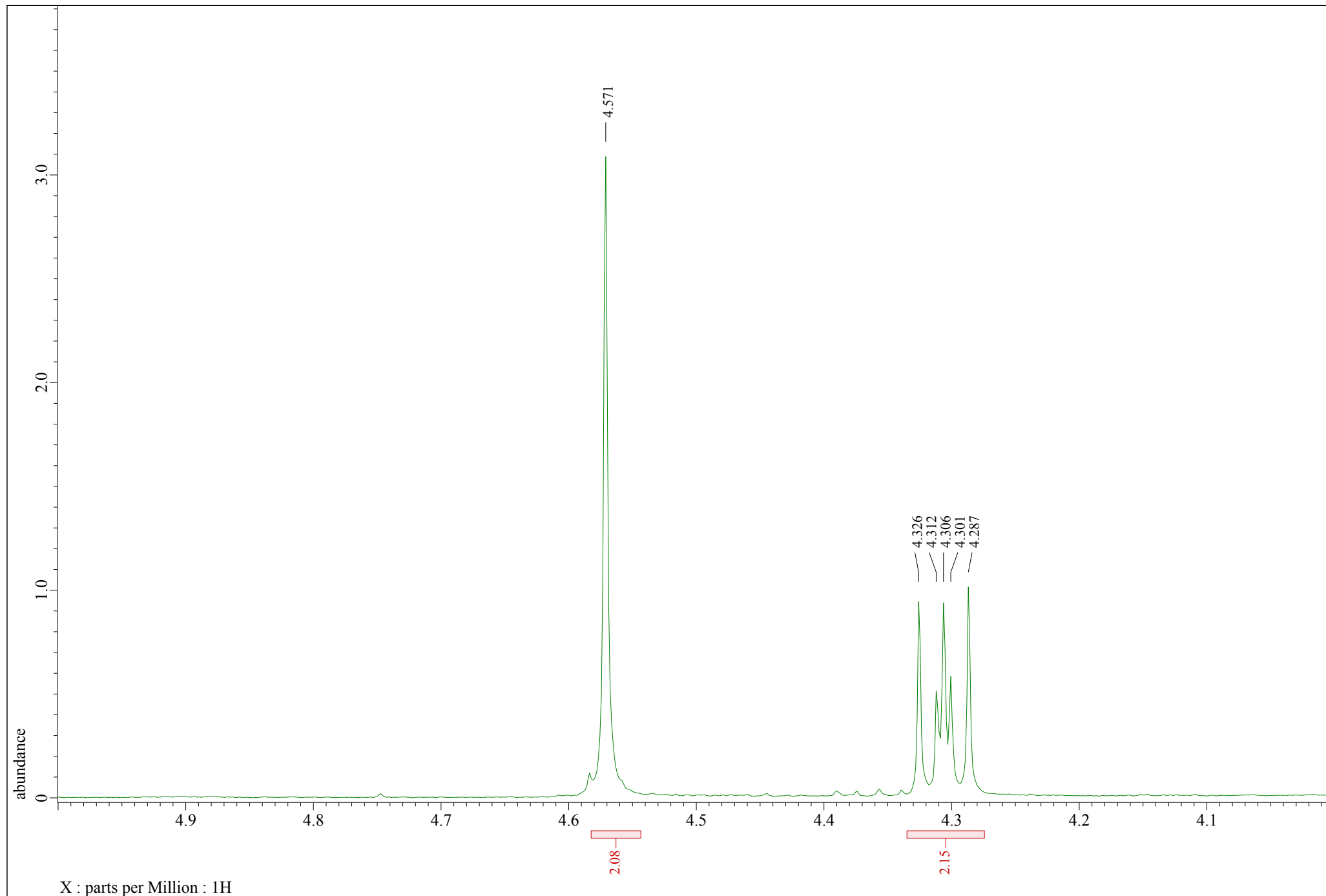

<sup>1</sup>H-NMR 5–7.5ppm

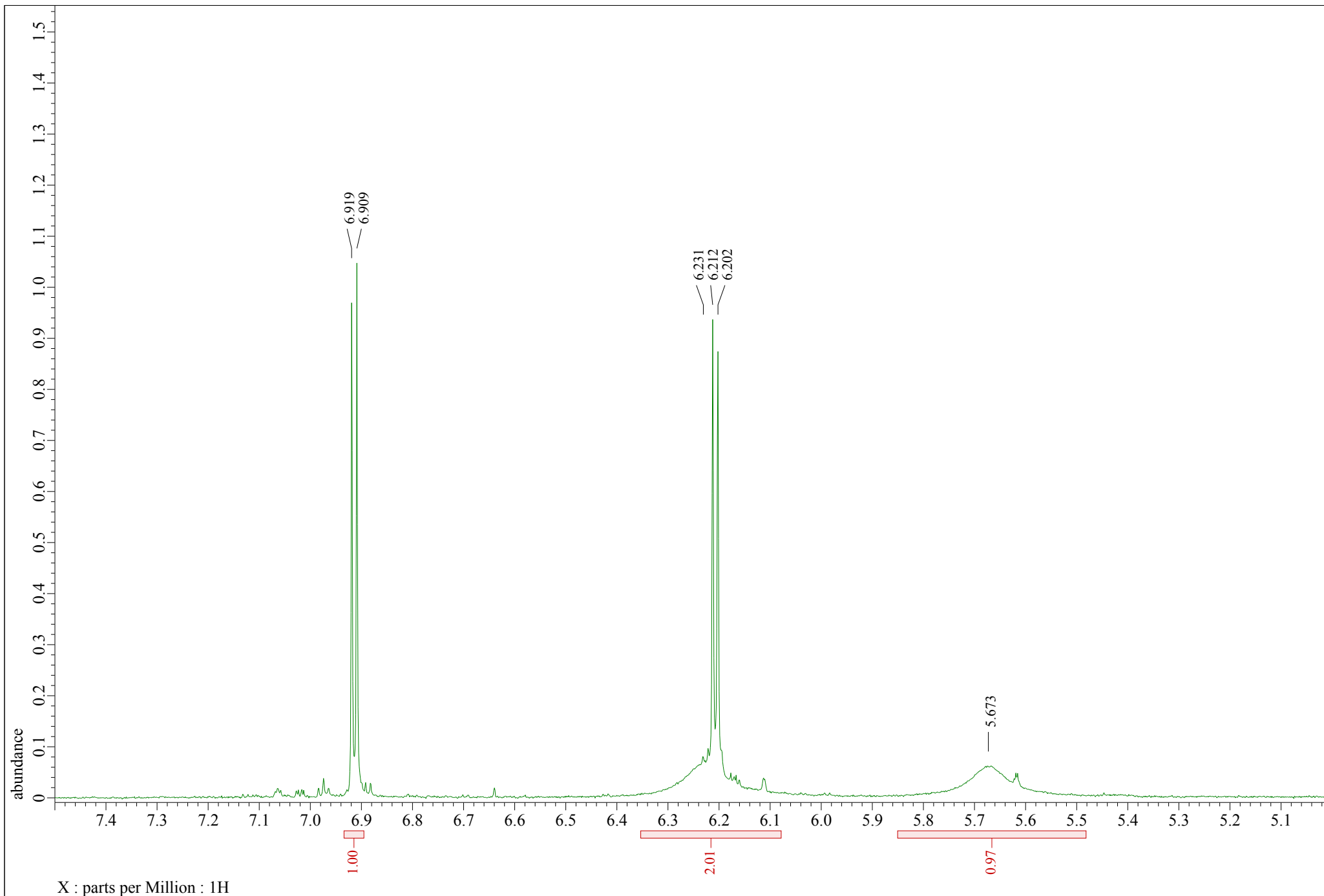

# <sup>13</sup>C-NMR

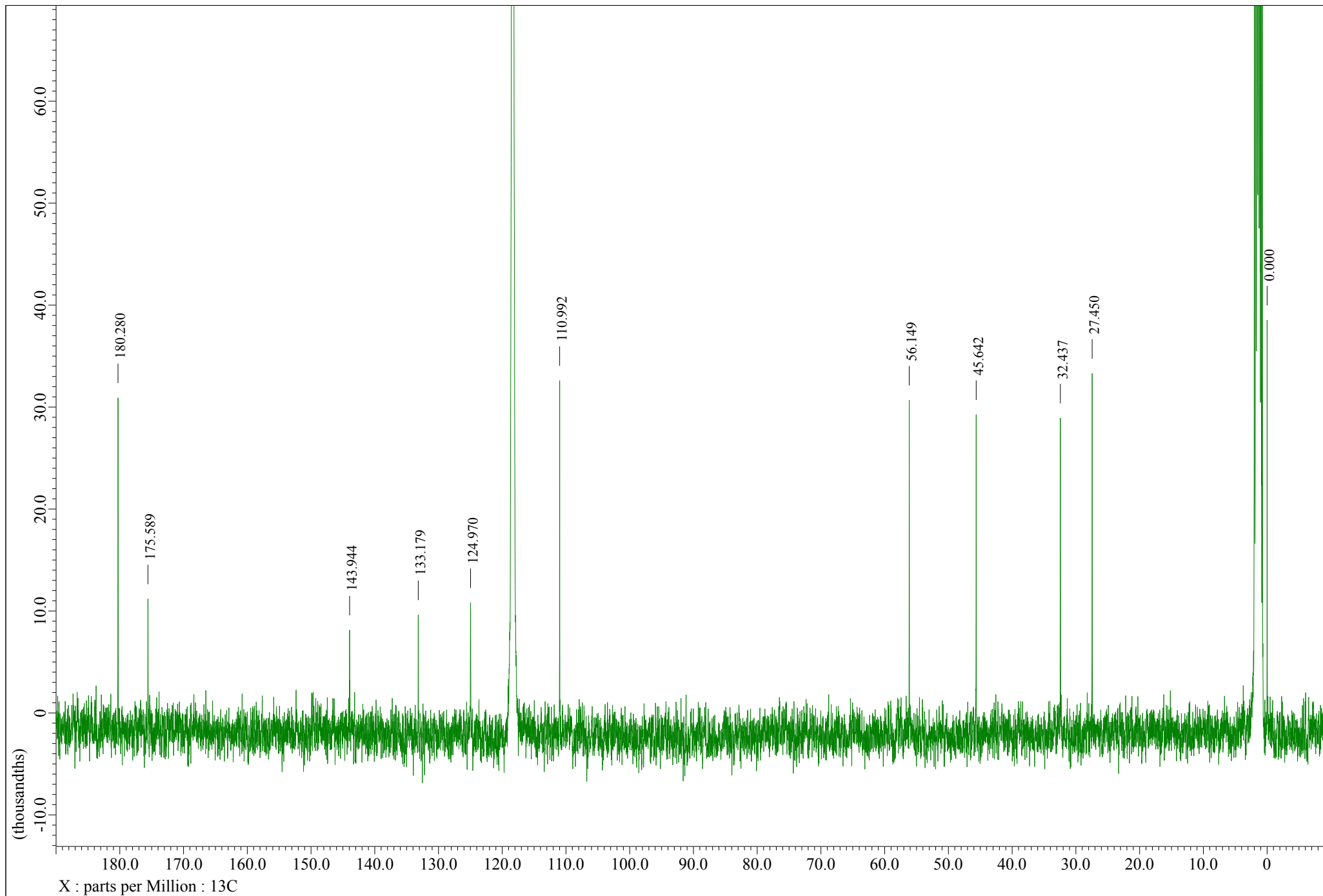

HMQC

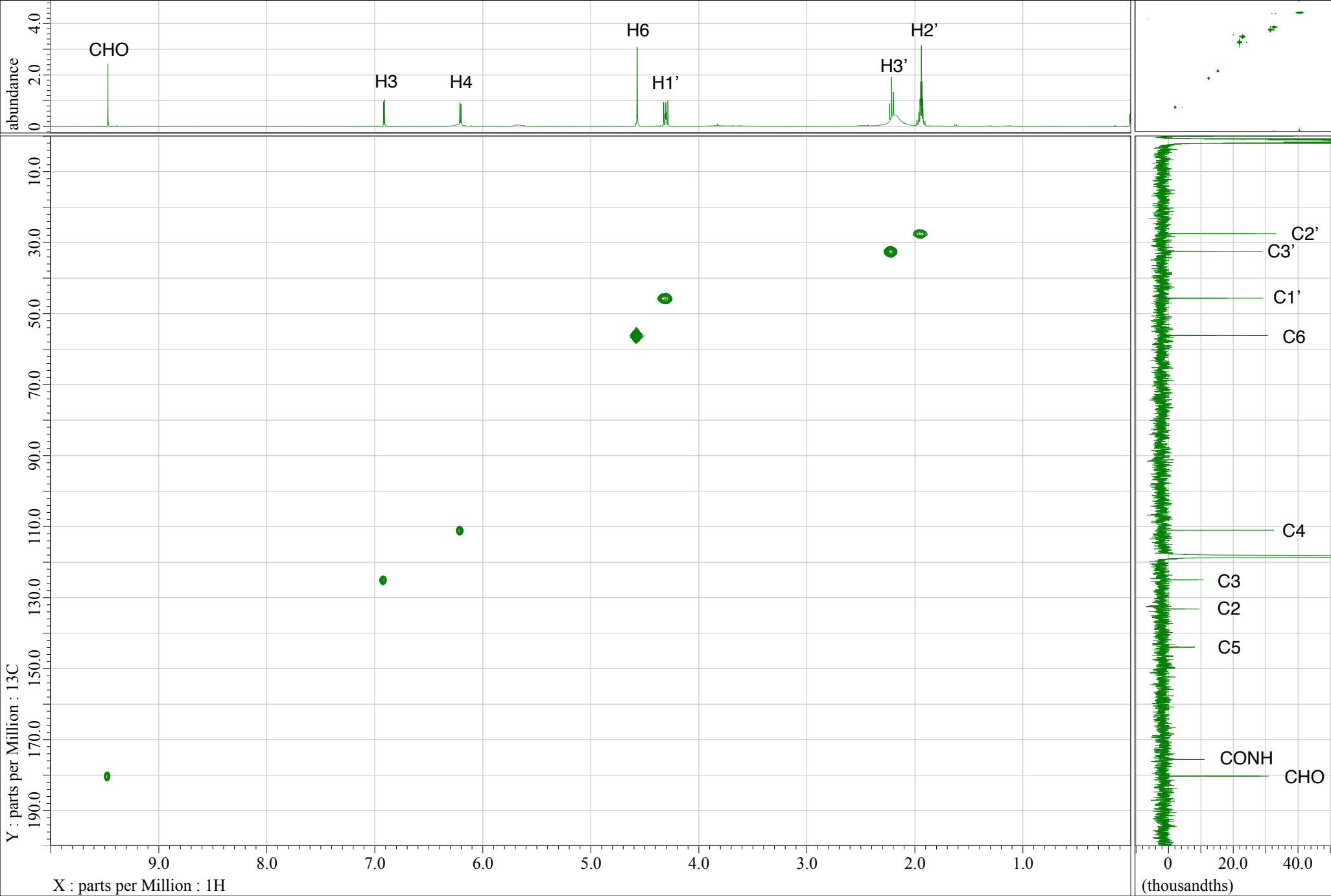

HMBC(4 Hz)

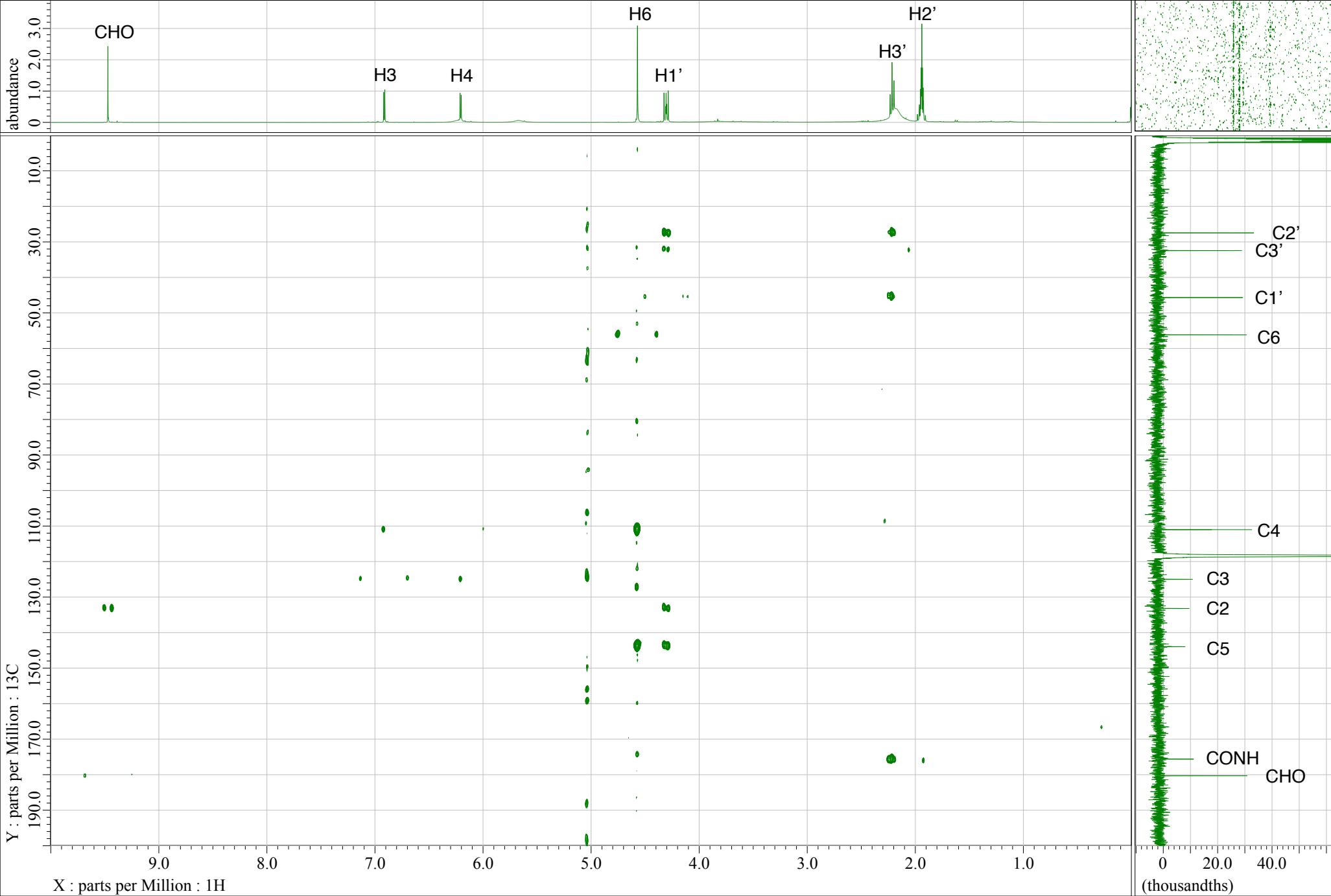

HMBC(8 Hz)

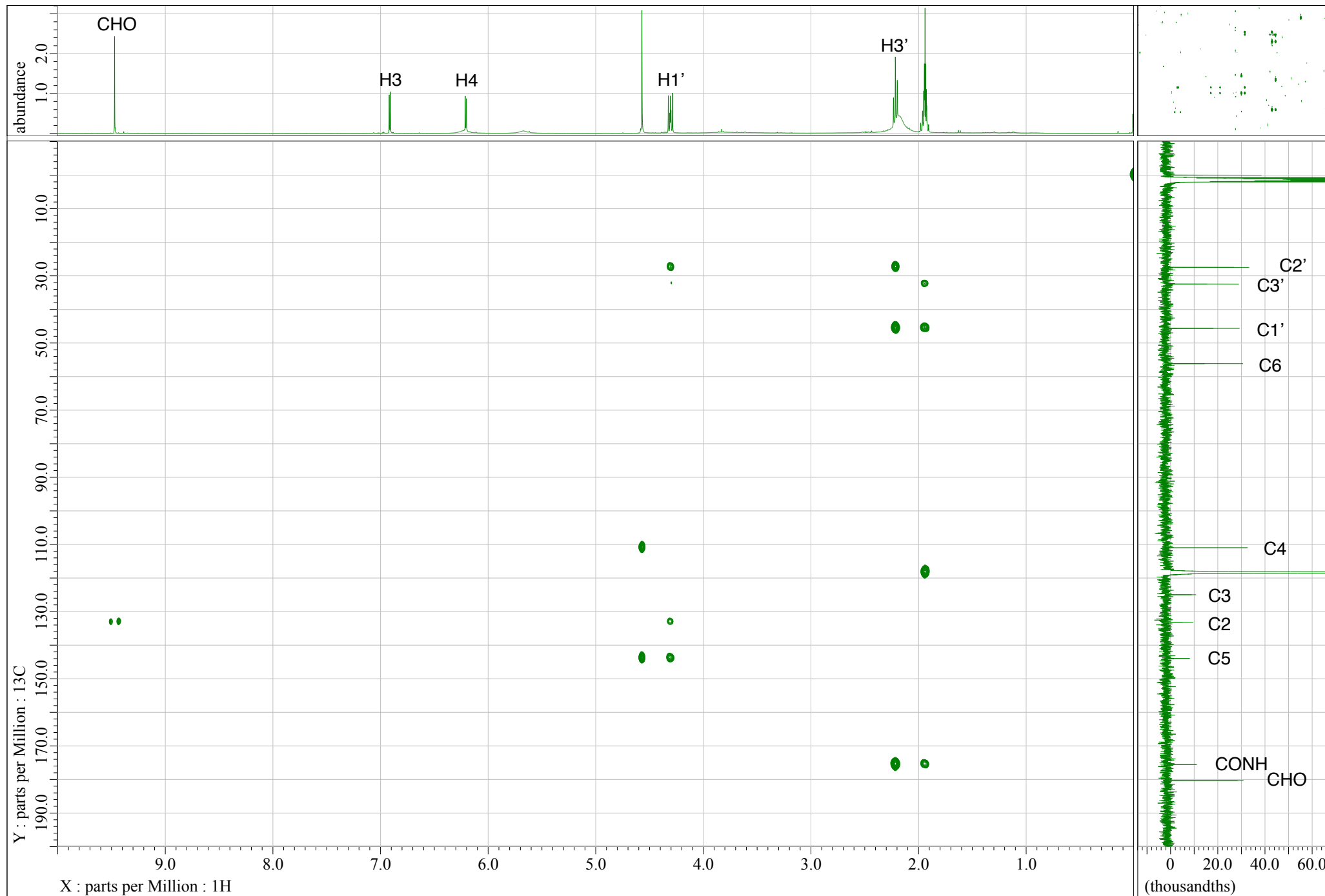

HMBC(12 Hz)

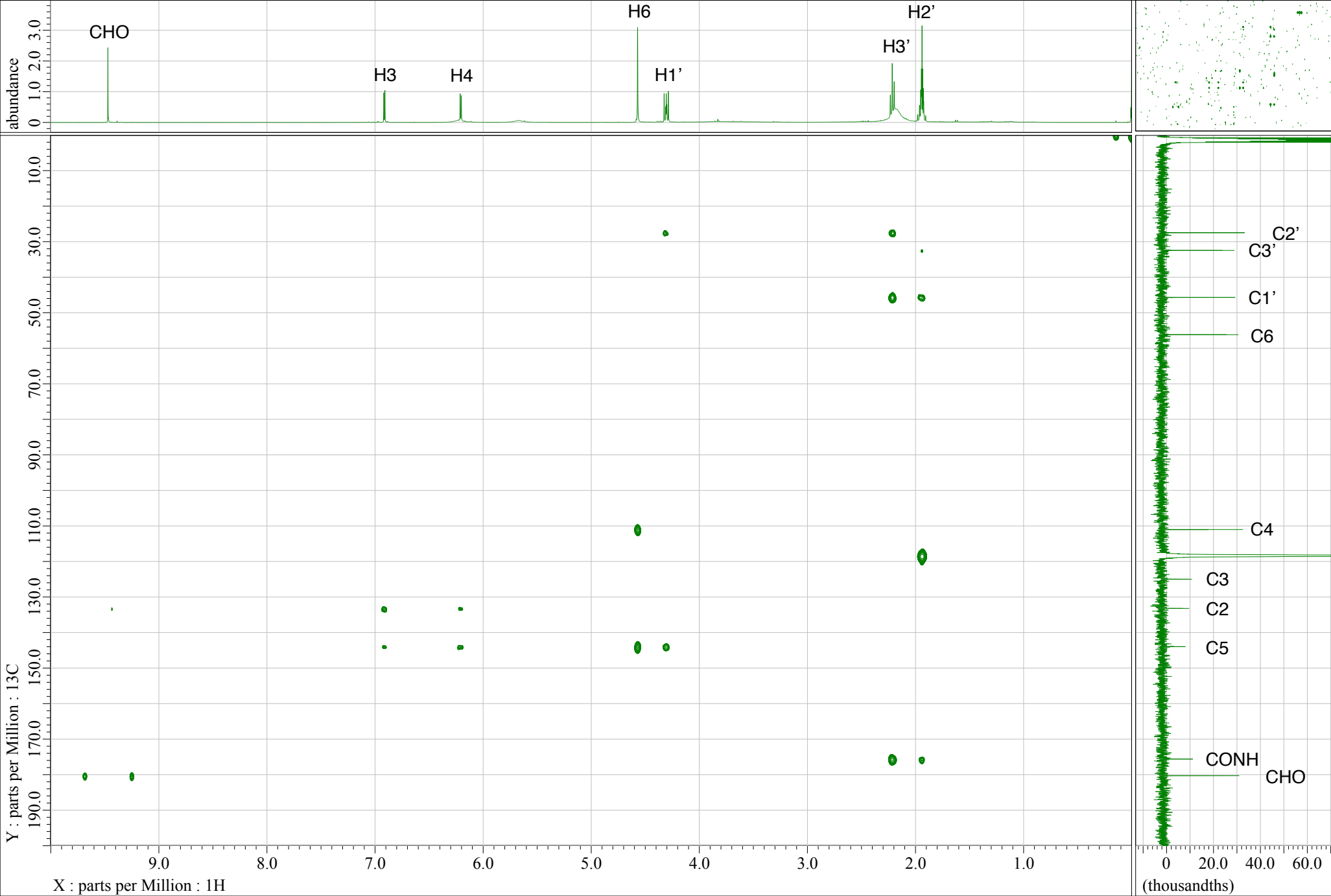

Supplement: Supplementary file 1 [file molecules-25-04879-s001.zip › NMR_data_r/Compound_II_NMR.pdf]

Compound-III 1H-NMR

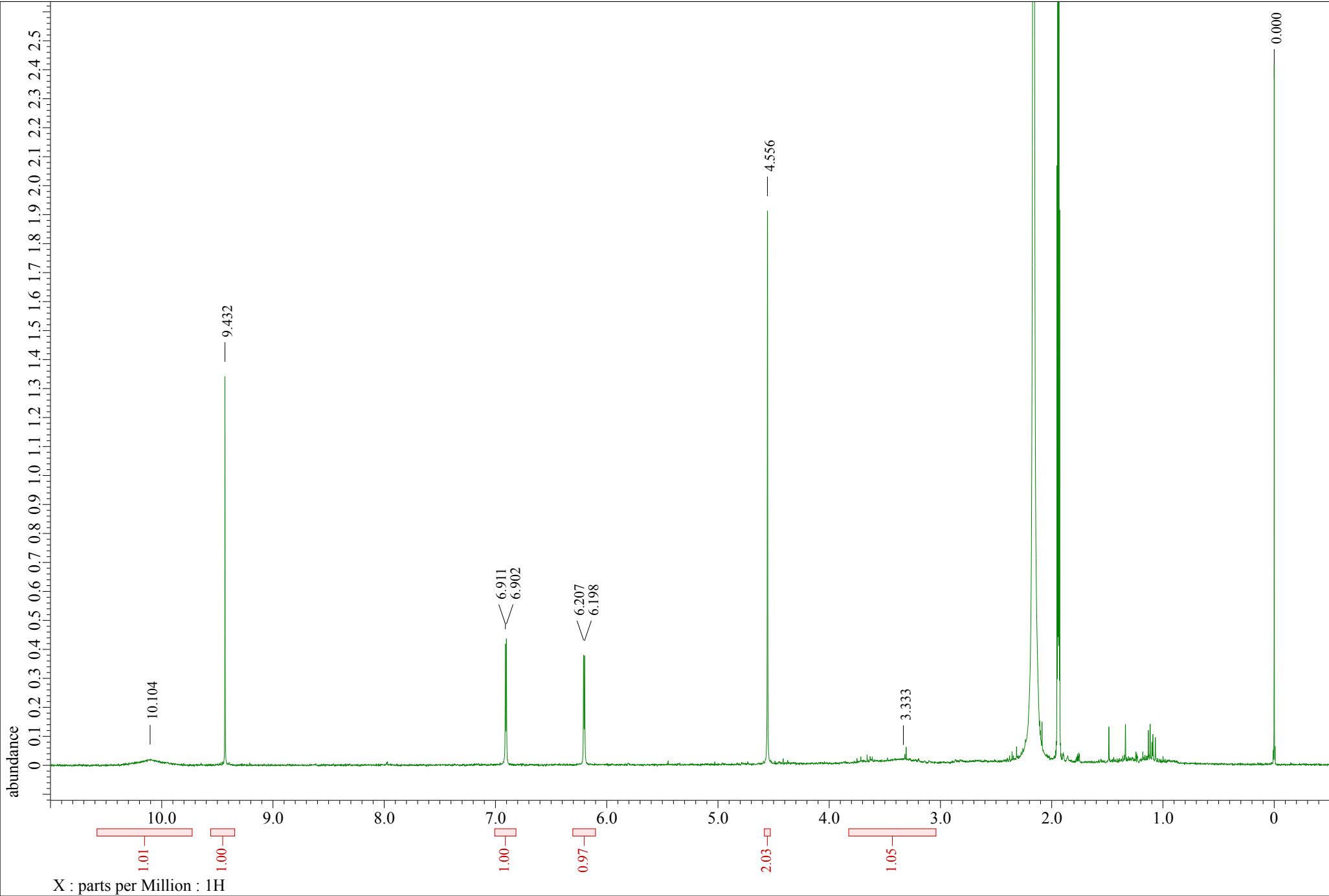

1H-NMR 6-7 ppm

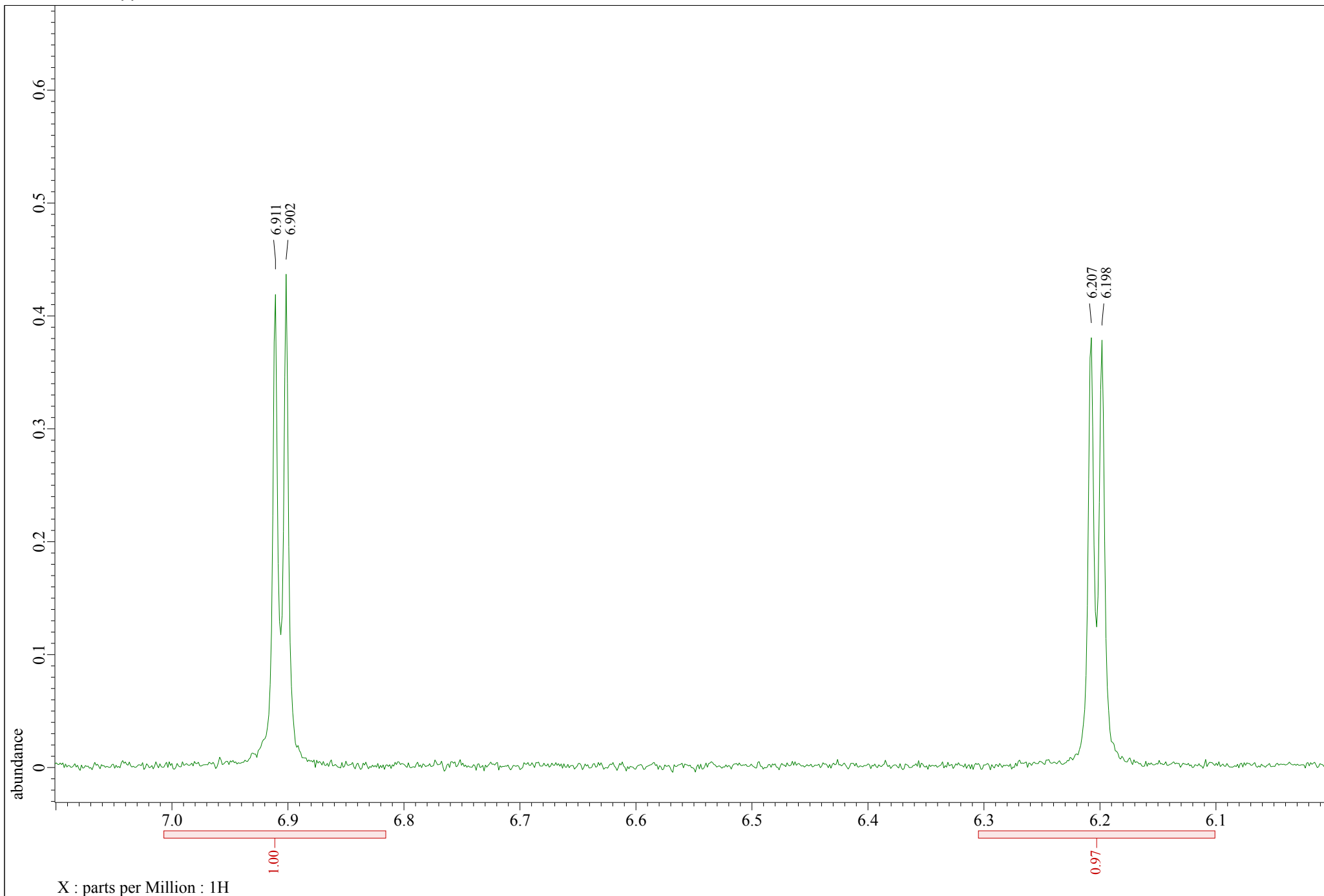

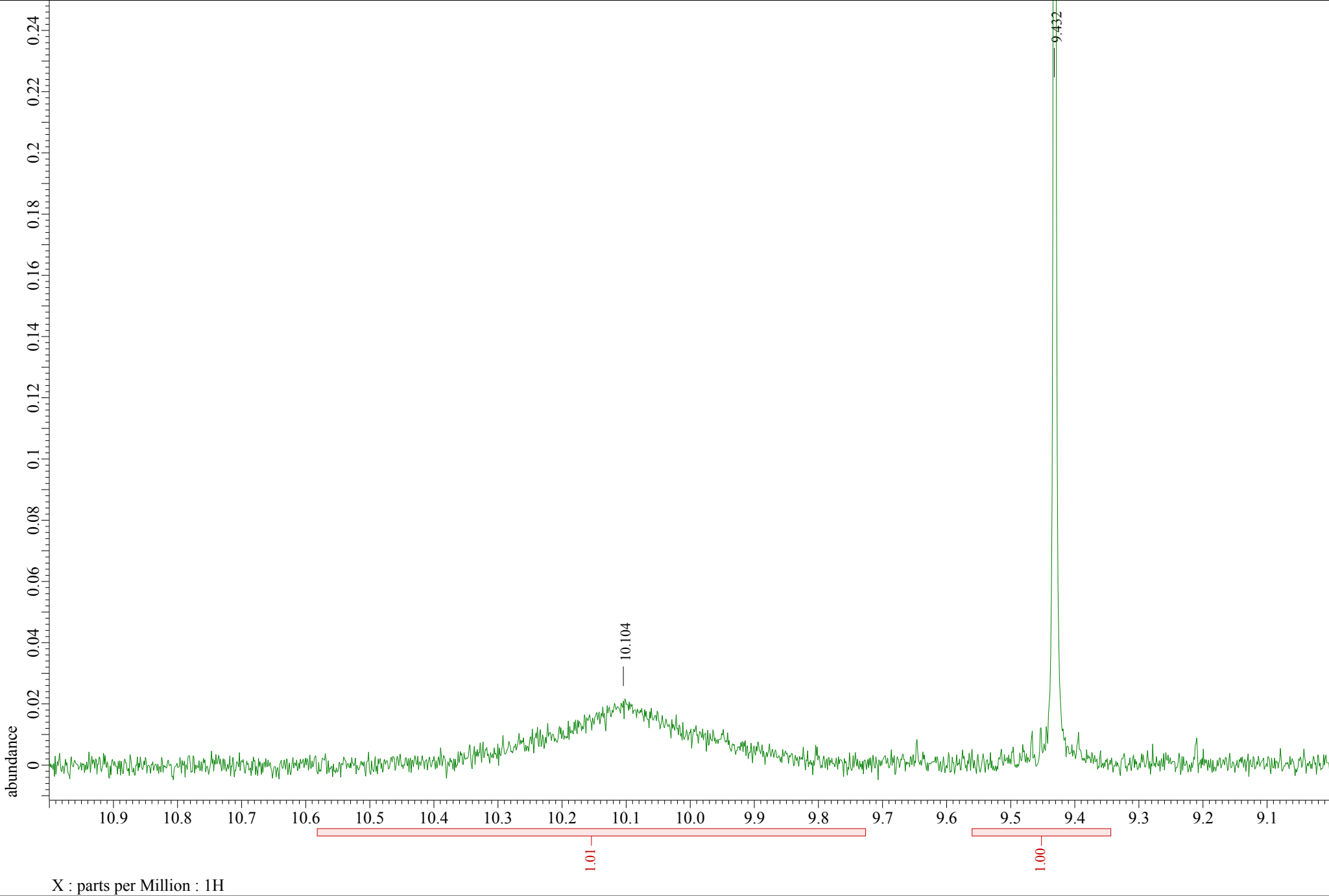

Supplement: Supplementary file 1 [file molecules-25-04879-s001.zip › NMR_data_r/Compound_III_NMR.pdf]
